# Prosthetic articulating spacers as a preferred option for two-stage revision arthroplasty in chronic periprosthetic joint infection

**DOI:** 10.1186/s42836-024-00288-6

**Published:** 2025-01-09

**Authors:** Jiamin Lin, Hongyan Li, Yang Chen, Haiqi Ding, Qijin Wang, Jianhua Lv, Wenbo Li, Wenming Zhang, Xinyu Fang

**Affiliations:** 1 Department of Orthopedic Surgery, the First Affiliated Hospital, Fujian Medical University, Fuzhou, 350005 China; 2 Department of Orthopedic Surgery, National Regional Medical Center, Binhai Campus of the First Affiliated Hospital, Fujian Medical University, Fuzhou, 350212 China

**Keywords:** Periprosthetic joint infection, Two-stage revision, Prosthetic spacer, Cement spacer

## Abstract

**Purpose:**

The study aimed to compare the infection control rates, mechanical complications, and functional outcomes between prosthetic and cement spacers in two-stage revision arthroplasty for chronic periprosthetic joint infection (PJI).

**Patients and methods:**

Data from patients treated for chronic PJI in our center from 2014 to 2023 were retrospectively collected and the patients were divided into the prosthetic spacer (PS) and cement spacer (CS) groups based on the type of spacer used for the first-stage surgeries. Data on patients’ demographics and clinical scores were harvested. Infection control rates and mechanical complications were compared between the two groups by using chi-square tests and log-rank analysis.

**Results:**

The study involved 113 cases, with a mean age of 64 ± 11.45 years (range, 31–88 years), with 48 cases in the PS group, 65 in the CS group, and all patients were followed up for at least 1 year (average 52.68 ± 26.07 months). Five patients in the PS group (10.42%) and six in the CS group (9.23%) developed recurrent infections, with no significant difference found in infection control rates (*P* = 0.833). The joint function score after the first-stage surgeries was higher in the PS group than in the CS group (*P* = 0.021). The incidence of mechanical complications, including dislocation, spacer fracture, and periprosthetic fracture, was significantly lower in the PS group than in the CS group (*P* = 0.024). The proportion of patients who underwent second-stage surgeries was lower in the PS group than in the CS group (58.3% vs 70.77%, *P* = 0.169).

**Conclusion:**

For most patients with chronic PJI, PS can be used as the preferred option for two-stage revision arthroplasty.

## Introduction

Periprosthetic joint infection (PJI) represents one of the main complications after joint replacement arthroplasty [1]. With joint replacement arthroplasty being increasingly performed, the incidence of PJI has also been steadily on the rise [2, 3], reportedly ranging from 0.5% to 1.9% and posing a growing burden on both patients and healthcare systems [4, 5].

Currently, the main treatment options for PJI include conservative treatment, debridement, antibiotics and implant retention (DAIR), one-stage revision, two-stage revision, joint fusion, and amputation [6], of which two-stage revision arthroplasty is considered the gold standard and the most widely used technique for the treatment of chronic PJI [7]. In the first-stage surgery, the joint prosthesis is removed, and the infected necrotic tissues and suspected infected tissue are thoroughly resected, followed by the implantation of spacers and a period of antibiotic treatment. When infection is controlled and after an average of 3 to 6 months, a second-stage surgery will be performed for further debridement and reimplantation of a new joint prosthesis. Antibiotic-containing polymethylmethacrylate (PMMA) bone cement is employed intraoperatively since it has been well documented and repeatedly validated after its original use by Buchholz and Engelbrecht (1970) [8] The implantation of an antibiotic-containing spacer plays an important role in two-stage revision arthroplasty, as it can release antibiotics directly into the joint to control local infection while maintaining the joint space and reducing soft tissue contracture to facilitate reimplantation of the prosthesis [9].

The commonly used types of cement spacers include static spacers, hand-made joint spacers, and prefabricated joint spacers [10]. Static spacers can immobilize the joint and increase stability, but long-term fixation can cause soft tissue contracture and fibrosis, leading to impaired joint movement function and difficulty in exposure during the second-stage surgeries, which adversely affect postoperative function [11]. Hand-made spacers currently occupy the mainstream position due to their convenience and low cost, but they are associated with a relatively high incidence of postoperative complications, including cement wear, spacer displacement, spacer fracture, joint instability, and dislocation [12]. Prefabricated cement spacers are designed to maximize strength and theoretically possess the best surface roughness to minimize wear debris on articular cement surfaces [13], but cement wear and fracture remain problems. Prosthetic spacers have greater strength and may, in theory, minimize the occurrence of cement debris and mechanical complications [14]. However, some studies have reported that prosthetic spacer surfaces can easily form bacterial biofilms in laboratory tests, which may increase the risk of recurrent infection after revision surgery [15].

Therefore, the purpose of this study was to compare the differences in infection control rates, postoperative function, and mechanical complications between the use of prosthetic spacers and all cement spacers in patients who underwent a two-stage revision for chronic PJI.

## Materials and methods

### Study design and data sources

**Fig. 1:**
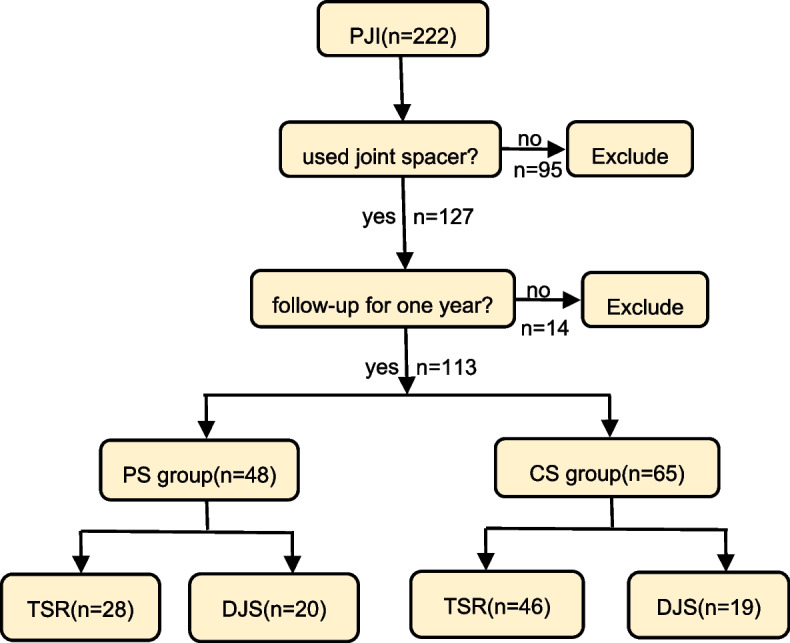
Flowchart showing inclusion and exclusion criteria

The study was approved by our Research Ethics Committee (Medical Research and Clinical Technology Application, Ethics Committee of the First Affiliated Hospital of Fujian Medical University [2021]404), and informed consent was obtained from the participants. A retrospective analysis was conducted on chronic PJI patients who underwent two-stage revision arthroplasty at our center between 2014 and 2023. The diagnosis of PJI was based on the diagnostic criteria formulated at the 2018 International Consensus Meeting on Orthopedic Infections [16]. The study divided patients into two groups in terms of the type of spacer used in the first-stage surgeries: i.e., a prosthetic spacer (PS) group and a cement space (CS) group. The inclusion criteria were as follows: (i) confirmed diagnosis of Tsukayama IV PJI; (ii) use of antibiotic-containing spacer during the revision arthroplasty; (iii) regular follow-up lasting for at least one year. The exclusion criteria included: (i) patients lost to follow-up; (ii) diseases that may affect the study results, such as malignant tumors and major organ dysfunction; (iii) patients whose deaths were unrelated to PJI. Among the 222 screened PJI cases, 55 cases underwent one-stage revision, 40 cases were treated with DAIR, and 14 cases were lost to follow-up, these cases were excluded. Eventually, a total of 113 chronic PJI cases were included (Fig. 1), with a mean age of 64 ± 11.45 years (range 31–88 years). A total of 48 cases were in the PS group and 65 in the CS group, and all patients were followed up for at least one year (an average time of 52.68 ± 26.07 months).

Patient gender, age, body mass index (BMI), age-adjusted Charlson Comorbidity Index (aCCI), preoperative C-reactive protein (CRP), erythrocyte sedimentation rate (ESR), synovial fluid-white blood cell (SF-WBC), synovial fluid-polymorphonuclear (SF-PMN), the visual analogue scale for pain (VAS), Harris hip score (HHS), Knee Society score (KSS), and preoperative and postoperative joint function scores after the first- and second-stage surgeries, as well as the date and operation time of first- and second-stage surgeries, were recorded. The operation time was the time from the start of the skin incision to the end of skin suturing. To further analyze the impact of undergoing the second-stage surgeries on the postoperative outcomes of the cases in this study, the two patient groups were further divided into two sub-groups: the two-stage revision (TSR) group, which received the second-stage surgeries, and the destination joint spacer (DJS) group, which did not undergo the second-stage surgeries when data were collected.

### Surgical procedure

**Fig. 2:**
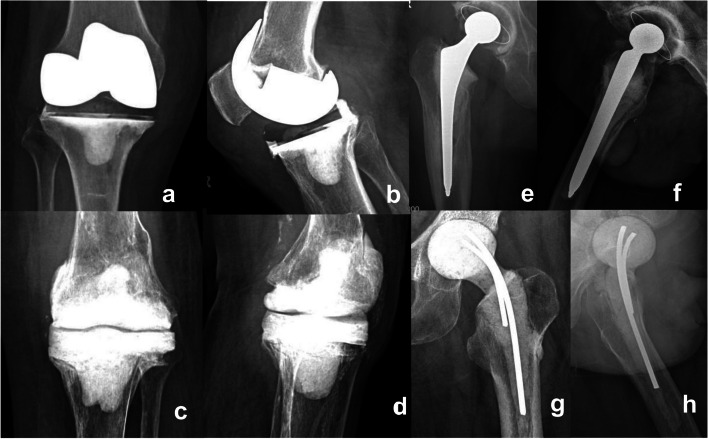
Joint spacer used for the first-stage revision

Revision arthroplasty was performed and joint spacers were produced by the same surgical team. Routine preoperative and intraoperative white blood cell (WBC) counts, polymorphonuclear (PMN) examinations, microbial cultures, and drug sensitivity tests were conducted. At least five specimens of prosthetic tissue around different parts were taken for microbial culture and pathological examination during surgery. After the prosthesis was removed, the infected necrotic tissue and suspected infected tissue were thoroughly resected, and the operated sites were repeatedly washed with hydrogen peroxide, iodophor, and saline. Then, antibiotic-containing PMMA bone cement joint spacers were produced. The antibiotics used in the spacers were selected according to the results of microbial culture and sensitivity tests. For patients whose microbial culture results were negative, intravenous vancomycin hydrochloride and meropenem were empirically used, with 2.0 g of vancomycin hydrochloride and 1.0 g of meropenem used for each 40 g of bone cement. Powdered antibiotics were added when bone cement was manually mixed and used in the dough stage. Two types of spacers were employed for the knee: type I (PS), in which the removed femoral condyle prosthesis was washed, soaked in iodophor solution for sterilization, and fixed with an antibiotic bone cement-coated sterilized femoral condyle prosthesis and a new polyethylene tibial pad (Fig. 2a&b), and type II (CS) which was made in situ using a sterile joint mold or hand-molded to make the all cement joint spacer (Fig. 2c&d). Two types of joint spacers were also used for the hip: type I (PS), which used a 105-mm femoral stem, a 28-mm femoral head as support on the femoral side, and a 32-mm cement-type acetabular cup on the acetabular side. Antibiotic-containing bone cement was applied and fixed (Fig. 2e&f). For Type II (CS), two Koehler needles with a diameter of 5 mm were used as supports and bent to about 130°, which were placed in the femoral head mold filled with antibiotic-containing bone cement. The distal end was coated with antibiotic-containing bone cement to make the spacer (Fig. 2g&h). After surgery, antibiotic therapy was administered. When infection was controlled, the joint spacer must be removed completely along with the necrotic granulation tissue, scar tissue, and cement fragments, and the operated sites were washed with a large volume of hydrogen peroxide, iodophor, and normal saline. Afterward, a new joint prosthesis was re-implanted.

### Postoperative treatment

Antibiotic sensitivity was determined based on postoperative drug sensitivity results. Antibiotics were intravenously given for 2–4 weeks and then orally for another 2–4 weeks. Patients were followed up regularly and tested for complete blood count, ESR, and CRP to monitor recurrent infection. Joint X-rays were performed regularly to observe for spacer loosening, subsidence, fracture, periprosthetic fractures, and joint dislocation. After antibiotic therapy was stopped for 4–6 weeks if there was no clinical recurrent infection, a second-stage surgery was performed.

### Postoperative evaluation

All patients were followed up regularly and reviewed after surgery to evaluate whether the infection had been cured. The infection cure was defined on the basis of the international multidisciplinary consensus of Delphi [17], which includes: (i) no sinus or pain in the wound, and no recurrent infection caused by the same pathogen; (ii) no additional surgical intervention due to infection after the replacement of the prosthesis; (iii) no deaths related to artificial joint infection, such as sepsis or necrotizing fasciitis. Functional recovery was evaluated by the HHS and KSS, and pain was assessed by the VAS. Postoperative mechanical complications were evaluated, including spacer fracture, periprosthetic fracture, and joint dislocation. The observation time for survival analysis was calculated in months, with the occurrence of recurrent infections or mechanical complications as outcome events. The survival time was from the day of surgery to the time the aforementioned outcome events were diagnosed. For patients without mechanical complications or recurrent infections, the survival time was from the day of the last surgery to the last follow-up. Censoring was used for deceased patients.

### Statistical analysis

All the statistical analyses were performed by using SPSS 25.0 (IBM, Armonk, NY, USA). For normally distributed data, comparisons were made using a *t*-test with a two-sided α = 0.05. For non-normally distributed data, comparisons were made using means (interquartile ranges) and the Mann–Whitney U tests. The rate was used to represent quantitative data, and chi-square tests were utilized for comparisons. A *P* ≤ 0.05 was considered statistically significant. Univariate log-rank analysis was performed to identify variables related to postoperative recurrent infections and mechanical complications, including spacer fracture, joint dislocation, and periarticular fractures. The survival analysis was performed using a multivariate Cox regression model.

## Results

### Demographic and clinical characteristics

The PS group included 48 cases, among which 28 underwent the second-stage surgeries, and 20 did not. The average duration, i.e., the time to reimplant after the last spacers lasted 267.20 ± 287.52 days. The average operation time of the second-stage surgeries was 178.65 min. The CS group included 65 cases, among which 46 received the second-stage surgeries, and 19 did not. The proportion of patients who underwent second-stage surgeries in the CS group was greater than that in the PS group (70.77% vs. 58.3%, *P* = 0.169). The average duration was 294.73 ± 356.84 days in the CS group, which was not significantly different from that in the PS group (*P* = 0.729). The average operation time of the second-stage surgeries lasted for 230.77 min in the CS group, which was significantly longer than that in the PS group (*P* < 0.001). The other demographic features of the two groups are presented in Table 1. There were no significant differences in the preoperative VAS score, HHS, KSS, and postoperative VAS score between the two groups (Table 2). The first-stage postoperative HHS and KSS of the PS group were significantly greater than those of the CS group (*P* = 0.021), and there was no significant difference in the joint function score after the second-stage surgeries between the two groups.

**Table 1:** Demographic information

	**PS group (*n* = 48)**	**CS group (*n* = 65)**	***P-*value**
Age	62.77 ± 10.34	64.91 ± 12.2	0.329
Gender			
Male	24	20	0.038
Female	24	45	
BMI (kg/m^2^)	25.08 ± 2.97	24.85 ± 3.35	0.707
aCCI (%)	3.39 ± 1.34	3.72 ± 1.69	0.271
CRP (mg/L)	41.36 ± 43.19	35.27 ± 37.46	0.468
ESR (mm/h)	71.58 ± 32.72	69.80 ± 37.08	0.809
SF-WBC (× 10^6^/L)	36,363.574 ± 48,081.34	50,917.03 ± 65,834.09	0.330
SF-PMN (%)	73.03 ± 27.63	79.15 ± 20.45	0.305

**Table 2:** Therapeutic evaluation

	**PS group (*n* = 48)**		**CS group (*n* = 65)**		***P-*value**		
	TSR (*n* = 28)	DJS (*n* = 20)	TSR (*n* = 46)	DJS (*n* = 19)	PS-TSR VS CS-TSR	PS-DJS VS CS-DJS	PS VS CS
Preoperative VAS	4.78 ± 1.33	4.90 ± 1.03	4.92 ± 1.49	4.93 ± 1.13	0.950	0.886	0.963
Postoperative VAS	3.03 ± 2.11	3.19 ± 2.12	2.64 ± 1.12	3.22 ± 2.01	0.519	0.668	0.412
Preoperative HHS	40.72 ± 8.61	42.72 ± 8.51	48.22 ± 10.44	42.62 ± 8.61	0.550	0.845	0.410
Postoperative HHS of first-stage	72.33 ± 12.13	69.93 ± 12.33	64.64 ± 18.60	63.55 ± 11.33	/	/	0.021
Preoperative KSS	37.66 ± 9.22	38.96 ± 9.22	44.88 ± 8.01	38.96 ± 9.22	0.220	0.201	0.203
Postoperative KSS of first-stage	68.45 ± 6.68	66.35 ± 6.18	60.50 ± 9.18	60.95 ± 6.18	/	/	0.022
Joint function score of second-stage	72.66 ± 7.33	/	69.03 ± 7.45	/	0.323	/	/
Reinfection	3	2	3	3	0.522	0.589	0.833
Spacer fracture	0	0	0	5	/	0.014	0.049
Periprosthetic fracture	2	2	3	2	0.918	0.957	0.901
Dislocation	0	1	3	5	0.168	0.065	0.047

### Comparison of infection control rates

**Fig. 3:**
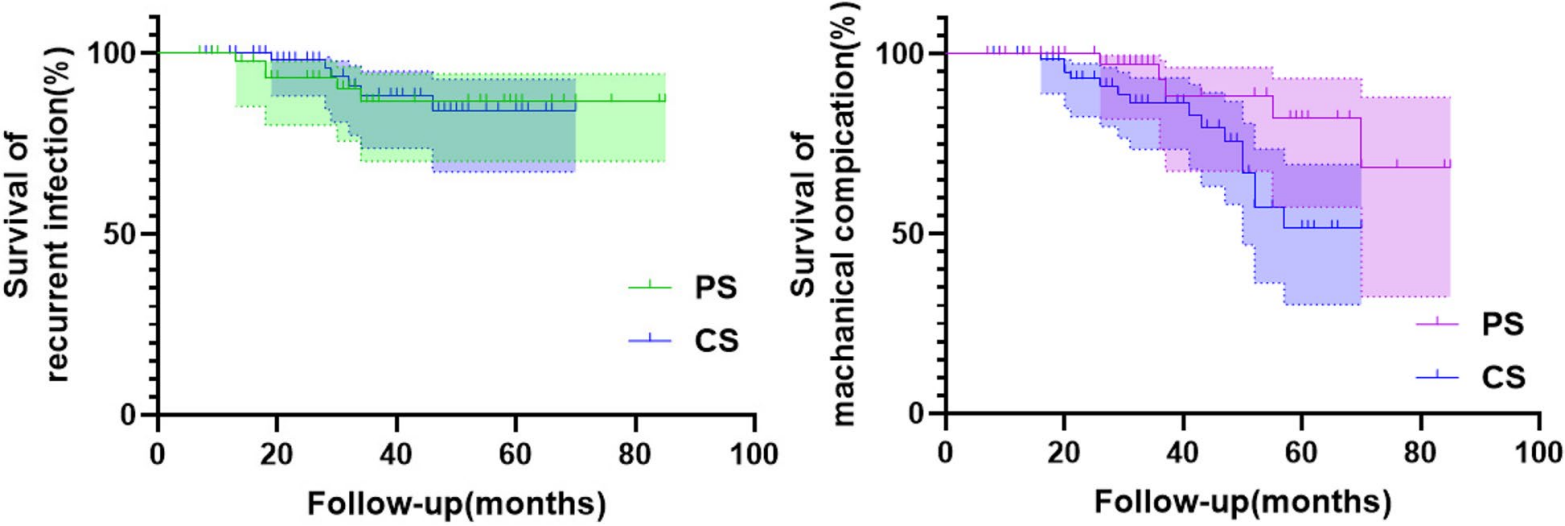
Log-rank test for Kaplan–Meier survival curve analysis of recurrent infection (left) and mechanical complications (right)

Five cases in the PS group developed recurrence (3 cases in duration, 2 cases after the second-stage surgeries). Among them, 3 cases of recurrence in duration were implanted with new spacers after debridement, and 2 cases of recurrence after the second-stage surgery underwent joint revision again. The CS group had 6 cases of recurrence (3 cases in duration and 3 cases after the second-stage surgery). Among 3 cases that experienced recurrence in duration, 2 cases underwent implantation of new spacers after repeated debridement, and in 1 case, a joint was fixed with an external fixation frame after removing the spacer. Among 2 cases that had recurrence after the second-stage surgeries, 2 cases underwent joint revision again, and 1 case was subjected to DAIR. There was no significant difference in infection control rates between the PS group (89.58%) and the CS group (90.77%) (*P* = 0.833). Information about the pathogenic microorganisms in the 113 patients is shown in Table 3. Further analysis revealed that the recurrent infections in patients with polymicrobial infections were significantly higher than those with monomicrobial infections (*P* = 0.049). Nonetheless, no significant associations were found between recurrence and patient age, comorbidities, spacer type, or the proportion of patients who underwent the second-stage surgery (Table 4). No significant difference was found in the survival curve of infection control rates between the PS group and the CS group (*P* = 0.872) (Fig. 3).

**Table 3:** Pathogenic microorganisms of the first-stage revision surgery

Variable	PS group (*n* = 48)	CS group (*n* = 65)
Culture negative	10	15
Culture positive	38	50
Staphylococcus	18	26
MRSA	1	2
*Pseudomonas aeruginosa*	1	3
*Listeria monocytogenes*	1	0
Corynebacterium striatum	2	1
*Candida albicans*	1	2
*Streptococcus gallinaceus*	0	1
*Bacteroides fragilis*	3	0
*Streptococcus dysgalactiae subsp*	2	1
*Streptococcus oralis*	0	1
*Klebsiella pneumoniae*	4	2
*Escherichia coli*	1	3
*Candida glabrata*	1	1
*Enterobacter cloacae*	0	1
*Streptococcus viridans*	0	1
*Corynebacterium urealyticum*	0	1
*Bacillus subtilis*	0	2
*Alcaligenes faecalis*	0	1
*Burkholderia cepacia*	1	0
*Rothia dentocariosa*	1	1
*Salmonella enterica* subsp	1	0

*MRSA* methicillin-resistant *Staphylococcus aureus*

**Table 4:** Univariate log-rank analysis of recurrent infection

Variable	Recurrent	Cure	*P*-value
**Comorbidity**			
Yes	7	68	0.861
No	4	34	
**Age**			
≥ 65	6	50	0.716
< 65	5	52	
**Spacer type**			
PS	5	43	0.644
CS	6	59	
**BMI**			
≥ 25	4	41	0.906
< 25	7	06	
**Underwent 2nd-stage surgeries**			
Yes	5	69	0.096
No	6	33	
**Polymicrobial infections**			
Yes	4	12	0.049
No	7	90

### Comparison of mechanical complications

The recognized mechanical complications of joint spacers include instability, dislocation, spacer fracture, and periprosthetic fracture. Spacer fracture is associated with subsequent instability and local soft tissue damage. Both groups in this study underwent regular postoperative follow-up and X-ray review to assess the occurrence of mechanical complications (Table 2). In the PS group, 4 cases developed periprosthetic fracture (2 cases in duration), and 1 case suffered from dislocation without spacer fracture. In the CS group, there were 5 cases of periprosthetic fracture (2 cases in duration), 5 cases of spacer fracture (all in duration), and 8 cases of dislocation (6 cases in duration, including 3 cases concurrent with spacer fracture and periprosthetic fracture). The number of mechanical complications was significantly lower in the PS group than in the CS group (*P* = 0.024). Univariate log-rank analysis revealed that gender, age, BMI, spacer type, and the presence of comorbidities may affect the incidence of mechanical complications in patients with chronic PJI (Table 5). The Kaplan–Meier survival regression curve showed that the survival curve of patients with mechanical complications in the CS group was lower than that in the PS group (*P* = 0.044) (Fig. 3). Upon screening, the factors that affect mechanical complications, in the order of relative hazard ratio from large to small, were spacer type, age, gender, presence of comorbidities in the multivariate COX regression model (Table 6), among which the spacer type was an independent risk factor (*P* = 0.030).

**Table 5:** Univariate log-rank analysis of mechanical complications

Variable	Complications	No complication	P value
**Gender**			
Male	9	35	0.681
Female	11	58	
**Comorbidity**			
No	6	32	0.860
Yes	14	61	
**Age**			
≥ 65	11	46	0.854
< 65	9	47	
**Spacer type**			
PS	5	43	0.044
CS	15	50	
**BMI**			
< 25	8	38	0.898
≥ 25	12	55

**Table 6:** Relative risk of multivariate analysis of mechanical complications

Variable	B	P	OR
Gender	0.066	0.862	1.068
Age	0.131	0.740	1.140
Comorbidity	− 0.046	0.907	0.955
Spacer type	0.858	0.030	2.359

## Discussion

This study revealed no significant difference in infection control rates between the PS group and the CS group. Although some studies reported that bacterial biofilm tended to form on the surface of PS, the formation of biofilms increased the resistance of bacteria to antibiotics and helped bacteria to escape the host immunity, thus causing various problems, such as persistent chronic clinical infection. However, there is currently no evidence that the use of polyethylene and metal prosthetic spacers results in a higher rate of biofilm formation than the use of all PMMA cement spacers [18]. From the perspective of prostheses, prosthetic surface structures that do not attract bacteria and the antibacterial coating of prosthetic surfaces can reduce bacterial biofilm formation, thus further reducing infection control rates.

In the PS group, there were 4 cases of periprosthetic fracture and 1 case of dislocation, without spacer fracture. In the CS group, there were 5 cases of periprosthetic fracture, 5 cases of spacer fracture, and 8 cases of dislocation. The number of mechanical complications in the PS group was significantly lower than that in the CS group (*P*= 0.024). This may be ascribed to the lower strength of the CS than that of the PS, the lower matching degree of the spacer interface, and the cement wear problems [19]. Therefore, the use of PS can prevent high rates of mechanical complications and postoperative failure rates caused by CS.

As new joint prostheses were used, there were no significant differences in functional scores after the second-stage surgeries between the two groups, while the HHS and KSS after the first-stage surgeries were significantly higher in the PS group than in the CS group. Our study showed that PS could achieve better postoperative function and provided an improved postoperative experience after the first-stage surgeries, thus lowering the proportion of undergoing the second-stage surgeries in the PS group compared to the CS group (58.3% vs. 70.77%, *P*= 0.169). Many authors reported that PJI posed a great financial burden on both patients and society at large [20, 21], therefore, the use of PS can not only attain better joint function after the first-stage surgery and reduce the risk of second-stage surgeries but also reduce the medical costs.

In 2022, Fei et al. [22] reported that there were no significant differences in infection control rates between metal polyethylene spacers and all-cement spacers in 47 cases, and metal polyethylene spacers outperformed all-cement spacers in terms of postoperative joint mobility, bone mass loss, and treatment cost of the metal polyethylene joint spacers. Kugelman et al. [23], in 2021, did not find any difference in the rate of recurrent infection and reoperation between these two types of spacers in 104 cases, while prosthetic spacers might provide significant comfort in the duration, and add no more risk. These findings were similar to our results, which also further illustrate the superiority of PS over CS in treating patients with chronic PJI.

## Limitations

This study still has some limitations: (i): this study was a single-center study with a small sample size, with only 48 PJI patients using PS and 65 PJI patients who used CS, (ii): the follow-up time was short, (iii): due to the epidemic of COVID-19, the proportion of patients who underwent the second-stage surgeries was significantly decreased during 2020–2022 and the duration was also significantly extended, which might affect the patients' recovery and statistics, to some extent.

## Conclusion

This retrospective study showed that when patients with chronic PJI underwent two-stage revision arthroplasty, the infection control rate was independent of the spacer type, while the incidence of mechanical complications was significantly lower in patients using PS than in those receiving CS, and the postoperative function was significantly better in PS group than in the CS group, which also reduced the risk of the second-stage surgeries and reduced the medical costs. Therefore, for most chronic PJI patients, PS can serve as a preferred option for the two-stage revision arthroplasty.

## Data Availability

All data and materials were in full compliance with the journal’s policy. The data were obtained from the Department of Orthopedic Surgery, the First Affiliated Hospital, Fujian Medical University. The datasets used during the current study are available from the corresponding author upon reasonable request.

## Abbreviations

PJI: Periprosthetic joint infection
DAIR: Debridement, antibiotics, and implant retention
PS: Prosthetic spacer
CS: Cement spacer
PMMA: Polymethylmethacrylate

## References

[1] Kheir MM, Tan TL, Shohat N, Foltz C, Parvizi J. Routine diagnostic tests for periprosthetic joint infection demonstrate a high false-negative rate and are influenced by the infecting organism. J Bone Jt Surg. 2018;100(23):2057–65. 10.2106/JBJS.17.01429. 10.2106/JBJS.17.01429 30516629

[2] Sloan M, Premkumar A, Sheth NP. Projected volume of primary total joint arthroplasty in the U.S., 2014 to 2030. J Bone Jt Surg. 2018;100(17):1455–60. 10.2106/JBJS.17.01617. 10.2106/JBJS.17.01617 30180053

[3] Khoshbin A, Stavrakis A, Sharma A, et al. Patient-reported outcome measures of total knee arthroplasties for post-traumatic arthritis versus osteoarthritis: a short-term (5- to 10-year) retrospective matched cohort study. J Arthroplasty. 2019;34(5):872-876.e1. 10.1016/j.arth.2019.01.022. 30745082 10.1016/j.arth.2019.01.022

[4] Gehrke T, Alijanipour P, Parvizi J. The management of an infected total knee arthroplasty. Bone Jt J. 2015;97-B(10_Supple_A):20–9. 10.1302/0301-620X.97B10.36475. 10.1302/0301-620X.97B10.36475 26430083

[5] Fang X, Wang Q, Yang X, et al. What is the appropriate extended duration of antibiotic prophylaxis after two-stage revision for chronic PJI? Bone Jt Res. 2021;10(12):790–6. 10.1302/2046-3758.1012.BJR-2021-0225.R1. 10.1302/2046-3758.1012.BJR-2021-0225.R1 PMC8696522 34894718

[6] Cai Y, Fang X, Huang C, et al. Destination joint spacers: a similar infection-relief rate but higher complication rate compared with two-stage revision. Orthop Surg. 2021;13(3):884–91. 10.1111/os.12996. 33768722 10.1111/os.12996 PMC8126900

[7] Chalmers BP, Mabry TM, Abdel MP, Berry DJ, Hanssen AD, Perry KI. Two-stage revision total hip arthroplasty with a specific articulating antibiotic spacer design: reliable periprosthetic joint infection eradication and functional improvement. J Arthroplasty. 2018;33(12):3746–53. 10.1016/j.arth.2018.08.016. 30236495 10.1016/j.arth.2018.08.016

[8] Buchholz HW, Engelbrecht H. Depot effects of various antibiotics mixed with palacos resins. Chirurg. 1970;41:511–5. 5487941

[9] Corró S, Vicente M, Rodríguez-Pardo D, Pigrau C, Lung M, Corona PS. Vancomycin-gentamicin prefabricated spacers in 2-stage revision arthroplasty for chronic hip and knee periprosthetic joint infection: insights into reimplantation microbiology and outcomes. J Arthroplasty. 2020;35(1):247–54. 10.1016/j.arth.2019.07.043. 31530462 10.1016/j.arth.2019.07.043

[10] Jaenisch M, Babasiz M, Ben Amar S, et al. Surgical technique and preliminary results of a moulded, mobile spacer for the treatment of periprosthetic joint infection of the knee. Oper Orthop Traumatol. 2023;35(3–4):163–9. 10.1007/s00064-023-00803-z. 37010531 10.1007/s00064-023-00803-z

[11] DeBoer DK. Comparison of traditional molded, first-generation premolded, and second-generation premolded antibiotic-loaded polymethylmethacrylate articulating spacers for treatment of chronic prosthetic joint infection of the knee. J Arthroplasty. 2020;35(3):S53–6. 10.1016/j.arth.2019.11.008. 32046833 10.1016/j.arth.2019.11.008

[12] Von Hertzberg-Boelch SP, Luedemann M, Rudert M, Steinert AF. PMMA bone cement: antibiotic elution and mechanical properties in the context of clinical use. Biomedicines. 2022;10(8):1830. 10.3390/biomedicines10081830. 36009376 10.3390/biomedicines10081830 PMC9404960

[13] Buller LT, Ziemba-Davis M, Meneghini RM. Complications and outcomes associated with a novel, prefabricated, articulating spacer for two-stage periprosthetic joint infection treatment. J Arthroplasty. 2021;36(12):3979–85. 10.1016/j.arth.2021.08.012. 34518057 10.1016/j.arth.2021.08.012

[14] Craig A, King SW, Van Duren BH, Veysi VT, Jain S, Palan J. Articular spacers in two-stage revision arthroplasty for prosthetic joint infection of the hip and the knee. EFORT Open Rev. 2022;7(2):137–52. 10.1530/EOR-21-0037. 35192512 10.1530/EOR-21-0037 PMC8897569

[15] Donlan RM. New approaches for the characterization of prosthetic joint biofilms. Clin Orthop. 2005;NA(437):12–9. 10.1097/01.blo.0000175120.66051.29. 10.1097/01.blo.0000175120.66051.29 16056020

[16] Schwarz EM, Parvizi J, Gehrke T, et al. 2018 international consensus meeting on musculoskeletal infection: research priorities from the general assembly questions. J Orthop Res. 2019;37(5):997–1006. 10.1002/jor.24293. 30977537 10.1002/jor.24293

[17] Diaz-Ledezma C, Higuera CA, Parvizi J. Success after treatment of periprosthetic joint infection: a Delphi-based international multidisciplinary consensus. Clin Orthop. 2013;471(7):2374–82. 10.1007/s11999-013-2866-1. 23440616 10.1007/s11999-013-2866-1 PMC3676607

[18] Gibon E, Córdova LA, Lu L, et al. The biological response to orthopedic implants for joint replacement. II: Polyethylene, ceramics, PMMA, and the foreign body reaction: BIOCOMPATIBILITY OF ORTHOPEDIC IMPLANTS. J Biomed Mater Res B Appl Biomater. 2017;105(6):1685–91. 10.1002/jbm.b.33676. 27080740 10.1002/jbm.b.33676 PMC5536115

[19] Yang FS, Lu YD, Wu CT, Blevins K, Lee MS, Kuo FC. Mechanical failure of articulating polymethylmethacrylate (PMMA) spacers in two-stage revision hip arthroplasty: the risk factors and the impact on interim function. BMC Musculoskelet Disord. 2019;20(1):372. 10.1186/s12891-019-2759-x. 31412841 10.1186/s12891-019-2759-x PMC6694660

[20] Moerenhout K, Steinmetz S, Vautrin M, Picarra S, Udin G, Borens O. Economic advantage of ‘self-made’ antibiotic-loaded spacer compared to prefabricated antibiotic-loaded spacer and spacer molds in two-staged revision arthroplasty. Acta Orthop Belg. 2021;87(3):557–62. 10.52628/87.3.24. 34808733

[21] Roof MA, Baylor JL, Bernstein JA, et al. Comparing the efficacy of articulating spacer constructs for knee periprosthetic joint infection eradication: all-cement vs real-component spacers. J Arthroplasty. 2021;36(7):S320–7. 10.1016/j.arth.2021.01.039. 33579629 10.1016/j.arth.2021.01.039

[22] Fei Z, Zhang Z, Wang Y, Zhang H, Xiang S. Comparing the efficacy of articulating spacers in two-stage revision for periprosthetic joint infection following total knee arthroplasty: all-cement spacers vs sterilized replanted metal-polyethylene spacers. Int J Gen Med. 2022;15:3293–301. 10.2147/IJGM.S354808. 35355797 10.2147/IJGM.S354808 PMC8959870

[23] Kugelman D, Roof M, Egol A, et al. Comparing articulating spacers for periprosthetic joint infection after primary total hip arthroplasty: all-cement versus real-component articulating spacers. J Arthroplasty. 2022;37(7):S657–63. 10.1016/j.arth.2021.12.008. 35210152 10.1016/j.arth.2021.12.008

